# The Antioxidants Glutathione, Ascorbic Acid and Uric Acid Maintain Butyrate Production by Human Gut Clostridia in The Presence of Oxygen *In Vitro*

**DOI:** 10.1038/s41598-020-64834-3

**Published:** 2020-05-07

**Authors:** Matthieu Million, Nicholas Armstrong, Saber Khelaifia, Elodie Guilhot, Magali Richez, Jean-Christophe Lagier, Gregory Dubourg, Eric Chabriere, Didier Raoult

**Affiliations:** 1 Aix Marseille Univ, IRD, AP-HM, MEPHI, Marseille, France; 2 IHU-Méditerranée Infection, Marseille, France

**Keywords:** Metabolomics, Microbiology, Gastroenterology, Pathogenesis

## Abstract

Uncontrolled oxidative stress, reported in *Salmonella* and HIV infections, colorectal cancer or severe acute malnutrition, has been associated with anaerobic gut microbiome alteration, impaired butyrate production, mucosal immunity dysregulation and disruption of host-bacterial mutualism. However, the role of major antioxidant molecules in the human body, such as glutathione, ascorbic acid and uric acid, has been neglected in this context. Here, we performed an *in vitro* metabolomics study of the 3 most odorous anaerobic microbes isolated from the human gut in our laboratory (*Clostridium sporogenes, Clostridium subterminale* and *Romboutsia lituseburensis*) when grown in anaerobiosis or in aerobiosis with these 3 antioxidant molecules via gas and liquid chromatography-mass spectrometry (GC/MS and LC/MS). There was no growth or volatile organic compound production in aerobic cultures without the 3 antioxidant molecules. In anaerobiosis, the major metabolic products of the bacteria were thiols, alcohols and short-chain fatty acid esters. The production of alkanes, cycloheptatriene and, paradoxically, increased butyrate production, was observed in the cultures grown in aerobiosis with the 3 antioxidant molecules. The qualitative shift suggests specific molecular mechanisms that remain to be elucidated. The increased production of butyrate, but also isobutyrate and isovalerate *in vitro* suggests that these 3 antioxidant molecules contributed to the maintenance and active resilience of host-bacterial mutualism against mucosal oxygen and uncontrolled oxidative stress *in vivo*.

## Introduction

Odorants are volatile organic compounds (VOCs) detected by the human nose that generate an olfactive signal and mainly include sulfur compounds (hydrogen sulfide, ammonium hydrogen sulfide and thiols (also named mercaptans)), low-molecular-weight carboxylic acids, also known as short-chain fatty acids (SCFAs, such as acetate, propionate or butyrate), aldehydes, amines and heterocyclic compounds. Odorants, according to their chemical class, combination and/or concentration, lead to good or unpleasant odors^1,2^. Among the several gut microbial odorants, butyrate is of particular interest to human health as it is the preferred energy source for colonic cells^3^. In addition, it inhibits inflammation and carcinogenesis, reinforces the colonic defense barrier, modulates gut epithelium permeability, promotes satiety, increases sodium and water reabsorption (anti-diarrheal agent) and reduces oxidative stress^4^. Production of butyrate by gut commensals contributes to immunity regulation of the host by inducing the differentiation of colonic and systemic regulatory T cells^5,6^ and has an important epigenetic effect on gut epithelial cells by inhibiting histone deacetylase (HDAC)^7,8^. More specifically, a lower butyrate-to-acetate ratio has been associated with nonalcoholic steatohepatitis (NASH), adenomatous polyps or colon cancer^9^.

On the other hand, we have recently identified a link between redox state and the microbiome^10^. That is, there is a link between redox potential, oxidative stress and human microbiota according to the oxygen tolerance of each species and the abundance of antioxidants in the environment. For the first time, we found a statistically significant association between fecal redox potential and metagenomic relative aerotolerant predominance^10^. In severe malnutrition, the lack of antioxidants and nutrients necessary for endogenous antioxidants and major liver peroxisomal and mitochondrial dysfunction results in major and uncontrolled oxidative stress^11,12^. In children with severe acute malnutrition, we have shown a major depletion of anaerobic microbes (intolerant to reactive oxidative species)^13^, consistent with the collapse of fecal butyrate in those who die^14^. This led us to rediscover that antioxidants allow for most anaerobes, including methanogenic archaea, to thrive in an oxidative (aerobic) environment^15–20^.

Serendipitously, we observed an absence of unpleasant odor when anaerobes were grown aerobically with antioxidants. To test what might explain the mitigation of the adverse smell, we selected 3 strains of the human gut isolated by microbial culturomics^21–23^ that produced the strongest smells and cultured them in our anaerobic culture laboratory: *Clostridium sporogenes, Clostridium subterminale* and *Romboutsia lituseburensis*. We compared the VOCs produced by these 3 strains grown under standard anaerobic conditions or in ambient air with the addition of antioxidants (glutathione, ascorbic acid, and uric acid)^15–17^ using gas and liquid chromatography coupled with mass spectrometry. We were surprised to see that the profile of VOCs was quantitatively but also qualitatively different. Indeed, the aerobic metabolic profile (available only with the 3 antioxidant molecules) was different from the anaerobic metabolic profile, which was confirmed by analysis of polar metabolites. The anaerobic metabolic profiles allowed for us to distinguish each species with perfect accuracy. Unexpectedly, butyrate production by the bacteria was increased in aerobiosis with medium supplemented with uric acid, ascorbate, and glutathione, suggesting a critical role of these antioxidant molecules in the maintenance of butyrate production at the gut microbial-mucosal interface.

## Results

The 3 bacterial strains did not survive, and there was no production of VOCs or SCFAs by the bacteria in aerobiosis without the 3 antioxidant molecules. Accordingly, we compared the amount and profile of VOCs, SCFAs and polar metabolites of cultures grown in anaerobiosis and in aerobiosis with ascorbate, glutathione and uric acid.

### Amount and diversity of volatile organic compounds

**Figure 1:**
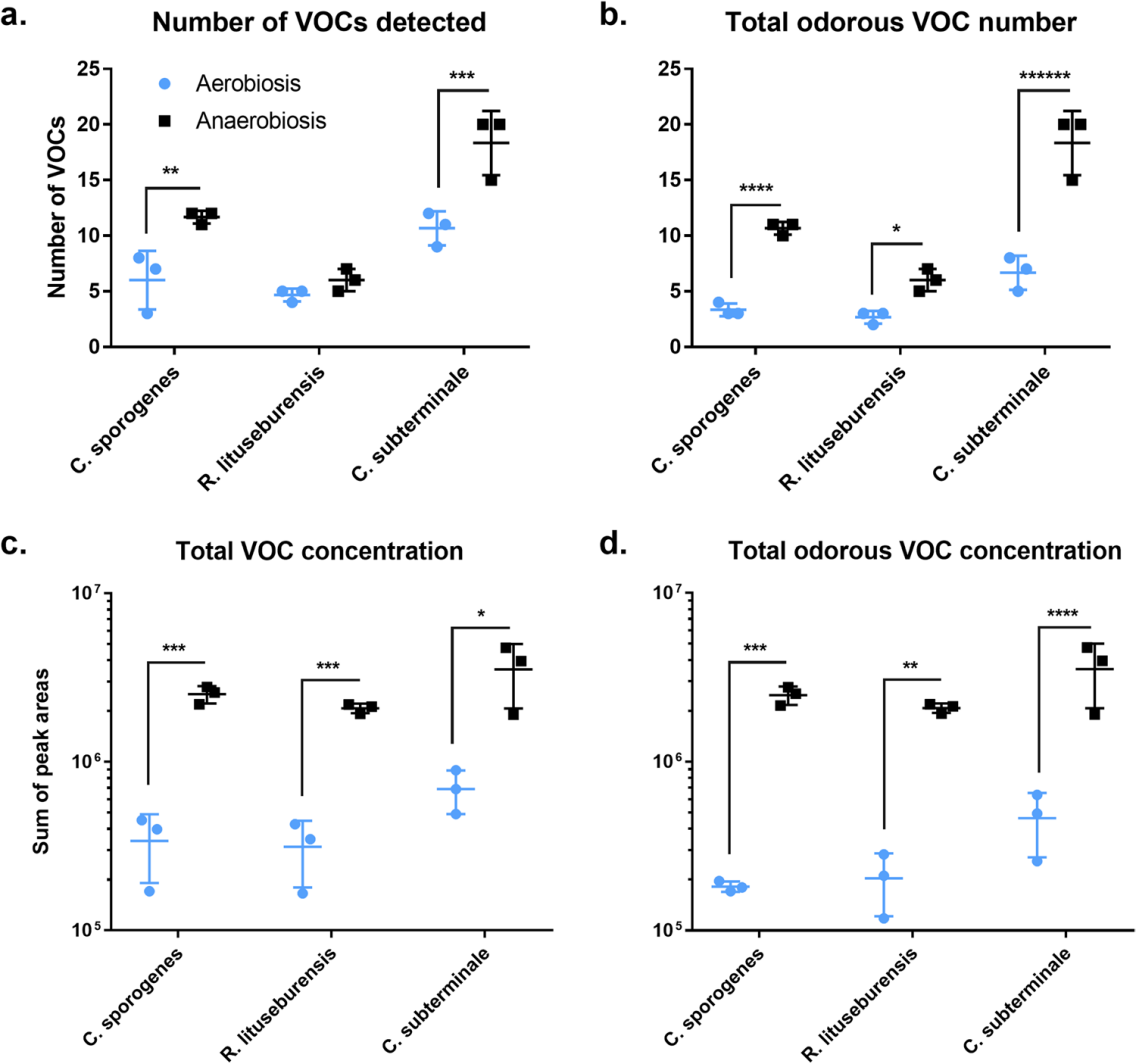
Diversity and total concentration of volatile organic compounds under antioxidant-based aerobic or anaerobic culture condition. VOCs: volatile organic compounds. The number (**a**) and total amount (**c**) of VOCs was higher in anaerobiosis for the 3 species and the difference was more significant considering only odorous VOCs (**b,d**). *p < 0.05, **p < 0.005, and so on.

The number and total amount of VOCs were higher in the cultures grown in anaerobiosis than in those grown in aerobiosis for all 3 species, and the difference was significant only for odorous VOCs (Fig. 1). Both the atmosphere and species determined the diversity and quantity of VOCs and odorous VOCs. However, the magnitude of the atmospheric effect was greater on VOC amounts than on their diversity. Indeed, the number of VOCs increased 1.2- to 1.8-fold (odorous VOCs: 2.0- to 3.7-fold), while the total amount increased 5.1- to 7.4-fold (7.7- to 13.6-fold) for bacteria grown in anaerobiosis when compared to those for bacteria grown in aerobiosis. The maximal atmospheric effect was observed for the total amount of odorous VOCs produced by *Clostridium sporogenes* (13.6-fold ratio, p-value < 0.00005, q-value < 0.0005).

### Chemical classes of volatile organic compounds

**Figure 2:**
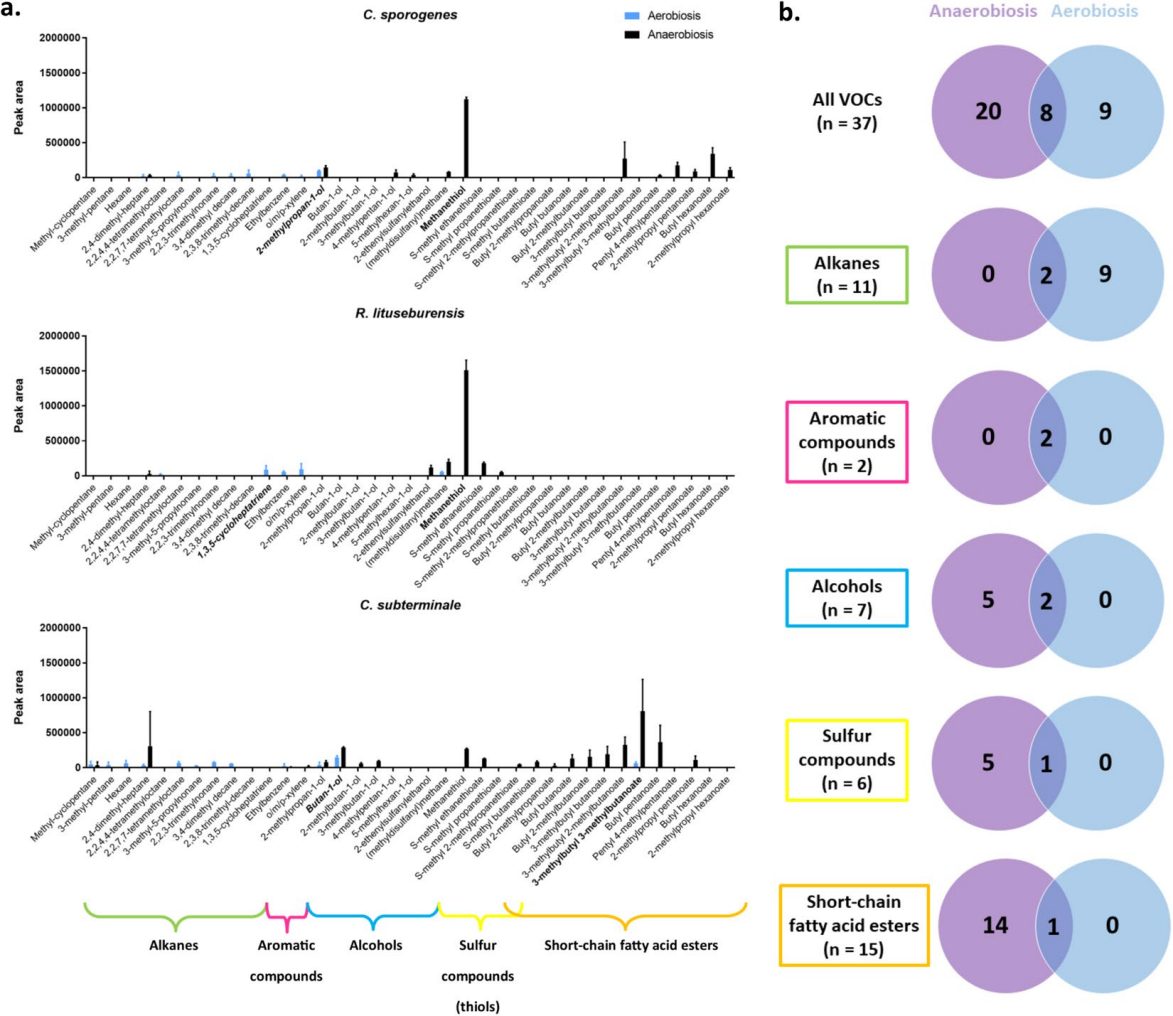
Diversity of volatile organic compounds according to species, chemical class and antioxidant-based aerobic or anaerobic culture condition. VOCs: volatile organic compounds. The 37 different VOCs produced by the 3 strains corresponded to 6 chemical classes, including 15 short chain fatty-acid ester (SCFAE), 7 sulfur compounds (thiols), 7 alcohols, 11 alkanes, 2 aromatic compounds and 1 alkene (**a,b**). 9 VOCs (alkanes) were detected only in aerobiosis (**b**). Anaerobiosis was associated with a specific and exceptional production of methanethiol for *R. lituseburensis* and *C. sporogenes* (**a**). The aerobic metabolic repertoire is different from the anaerobic one and is restricted suggesting a constrained metabolic behavior for VOC production redirected to the alkanes in the presence of oxygen.

The 37 different VOCs produced by the 3 strains corresponded to 6 chemical classes, including 15 short-chain fatty acid esters (SCFAEs), 7 sulfur compounds (including thiols), 7 alcohols, 11 alkanes, 2 aromatic compounds and 1 alkene (Fig. 2, Supplementary Table S1). By analyzing the number of VOCs detected for each culture condition regardless of the species, we found that the diversity of VOCs was reduced in cultures grown in aerobiosis since 17 VOCs were detected in those grown in aerobiosis and 28 in cultures grown in anaerobiosis (p < 0.05). Alkanes, aromatic compounds, alcohols, short-chain fatty acid esters, and sulfur compounds were detected in cultures grown both in aerobiosis and anaerobiosis (Fig. 2B). The alkene (1,3,5-cycloheptatriene) was detected only in cultures grown in aerobiosis. Sulfur alcohols and sulfur SCFAEs were detected only in cultures grown in anaerobiosis. Only one sulfur compound ((methyldisulfanyl) methane, commonly named dimethyl disulfide) and one SCFAE (3-methylbutyl 3-methylbutanoate) were detected in cultures grown both in aero- and anaerobiosis. This pattern remained unchanged over time, as determined by analyzing the VOCs produced after 48, 72 and 96 hours (Supplementary Fig. S1).

**Figure 3:**
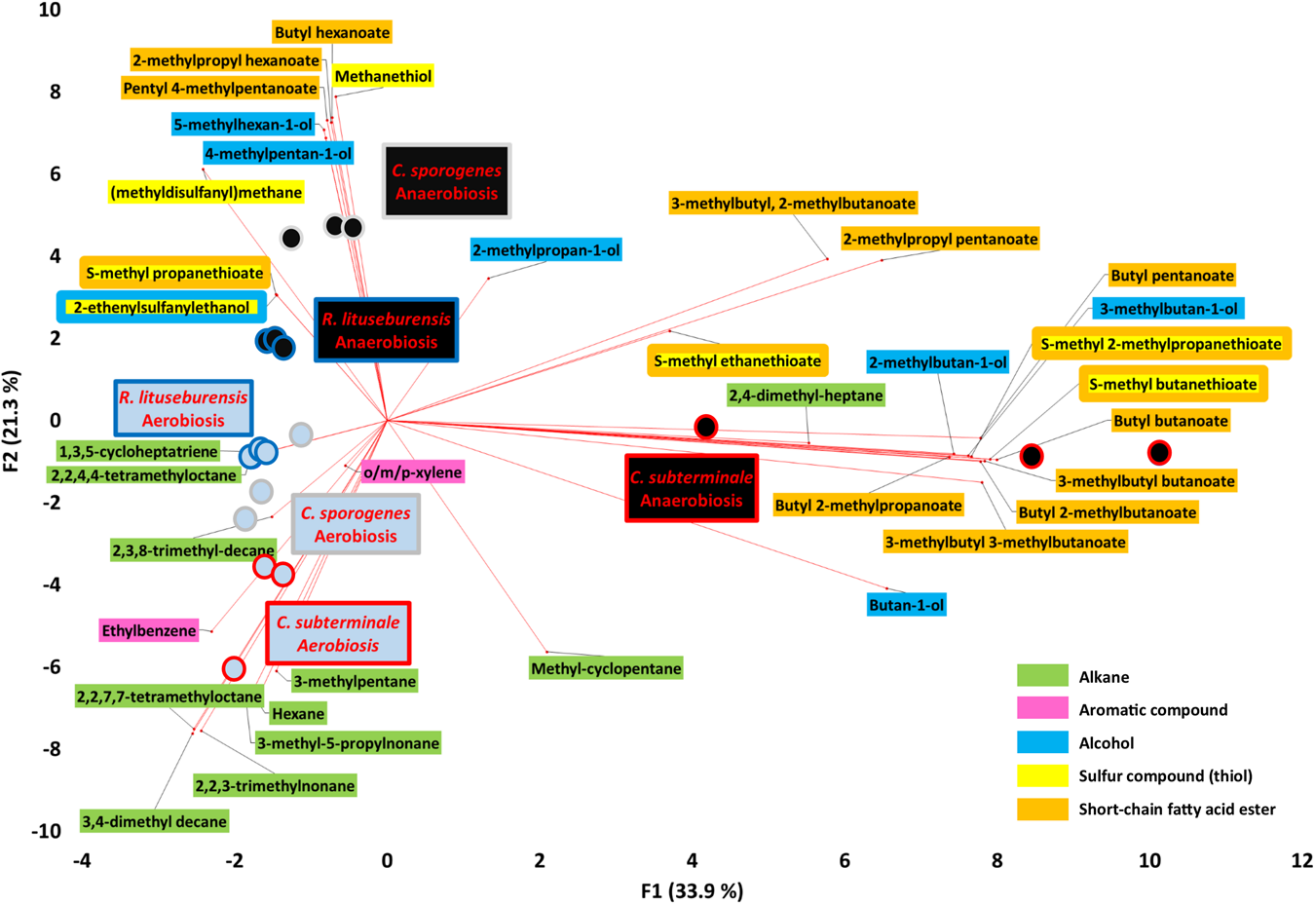
Biplot of the principal component analysis showing associations between species, culture conditions and volatile organic compounds. The VOCs associated with aerobic culture with antioxidants were clearly different and clustered compared to the VOCs produced anaerobically. In aerobiosis with antioxidants, the VOC patterns of the 3 bacterial strains were found similar and the VOCs produced belong to 2 classes, including 10 alkanes and 2 aromatic compounds (o/m/p-xylene and ethylbenzene). In contrast, VOC produced anaerobically exhibited a greater heterogeneity. This suggests that there is not only a quantitative change between the atmosphere but that a specific and unexpected aerobic metabolic repertoire can be observed when the 3 *Clostridia* species are grown in aerobiosis with antioxidants. Principal component analysis performed using XLSTAT v19.1.

This unexpected metabolic switching was confirmed by principal component analysis (Fig. 3). Indeed, the VOCs associated with aerobic culture with glutathione, ascorbic acid, and uric acid were clearly different and clustered compared to the VOCs produced anaerobically. In cultures grown in aerobiosis with the 3 antioxidant molecules, the VOC profiles of all 3 bacterial strains were found to be similar, and the produced VOCs mainly belong to 2 classes, including 10 alkanes and 2 aromatic compounds (o/m/p-xylene and ethylbenzene). In contrast, VOCs produced anaerobically exhibited greater heterogeneity. *C. sporogenes* and *R. lituseburensis* were grouped together, whereas *C. subterminale* was clearly different. This contrasts with the phylogenetic analysis (Supplementary Fig. S2). However, despite this heterogeneity, only one alkane was associated with anaerobic culture (2,4-dimethyl-heptane by *Clostridium subterminale*), while all short-chain fatty acid esters, thiols and alcohols were associated with anaerobiosis (Figs. 2, 3). This suggests that there was not only a quantitative change in the atmosphere but that a specific and unexpected aerobic metabolic profile can be observed when the 3 *Clostridia* species are grown in aerobiosis with antioxidants. The aerobic metabolic profile was entirely different from that of the anaerobic cultures.

### Limited metabolic repertoire of bacteria grown in aerobiosis

**Figure 4:**
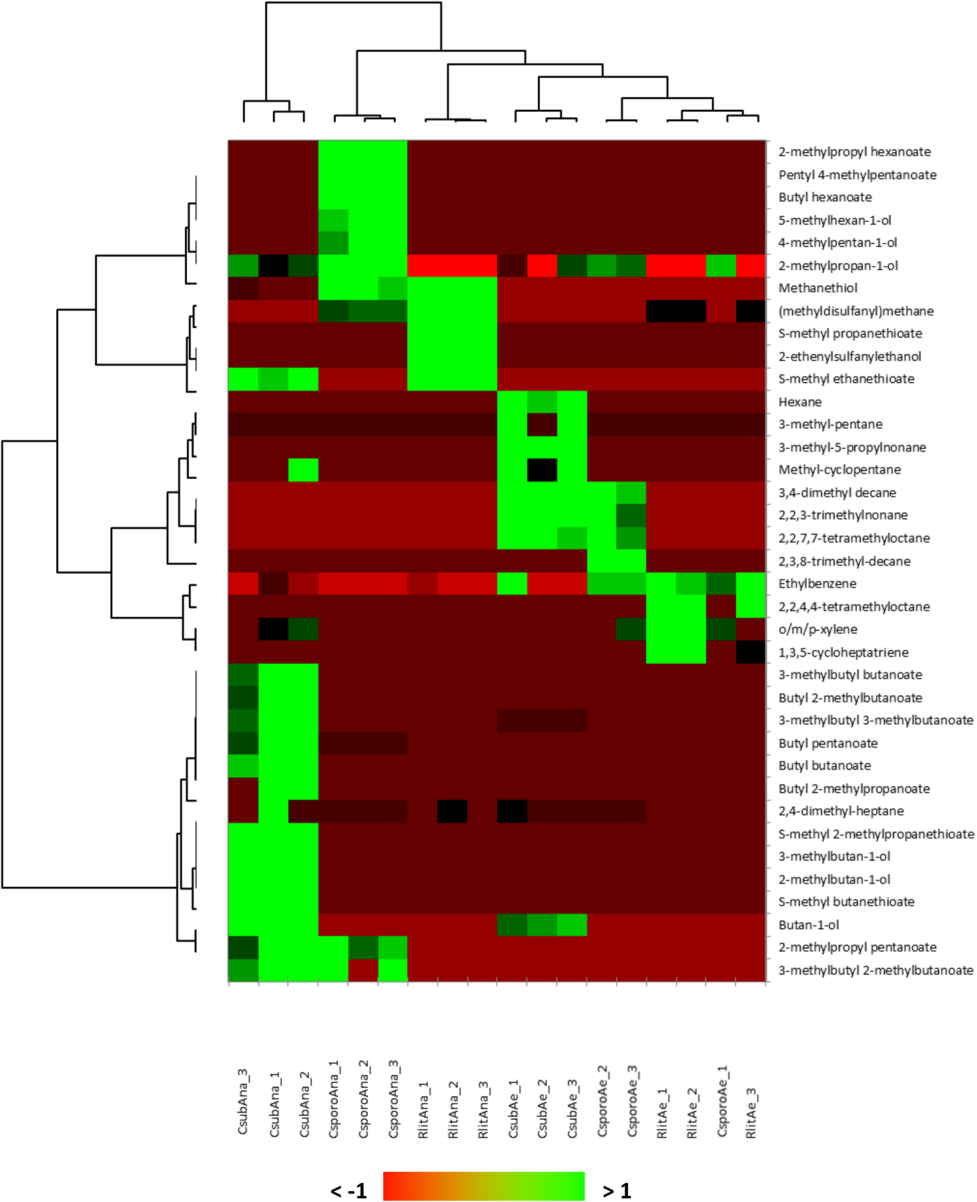
Species-specific VOC profile in anaerobiosis than in aerobiosis. A heatmap unsupervised analysis confirmed the dichotomic metabolic switching based on VOCs production since the atmosphere was the main discriminant factor and had a larger effect that species. Moreover, the accuracy of species identification was better in anaerobiosis (branch length between species was longer in anaerobiosis than in aerobiosis with an aerobic classification error: *C. sporogenes* among *R. lituseburensis –* perfect classification in anaerobiosis). The better species resolution in anaerobiosis was confirmed by discriminant analysis with 87.5% correct results in anaerobiosis versus 75% in aerobiosis after cross-validation. Heatmap unsupervised analysis performed using XLSTAT v19.1. Each experiment was replicated 3 times.

By analyzing the amount of each VOC produced by each species (Fig. 2, Supplementary Fig. S3, Supplementary Table S1), we observed that anaerobiosis was associated with a specific and exceptional production of methanethiol for *R. lituseburensis* (7.5-fold higher than the second highest VOC, (methyldisulfanyl)methane, commonly called dimethyl disulfide) and *C. sporogenes* (3.2-fold higher than the second highest VOC produced, butyl hexanoate). For *C. subterminale*, 3-methylbutyl 3-methylbutanoate was the most anaerobically produced VOC (Fig. 2 and Supplementary Fig. S3). We observed that *R. lituseburensis* produced neither SCFAEs or alcohol and that *C. sporogenes* produced no sulfur SCFAEs regardless of the atmosphere. In contrast, *C. subterminale* produced members of all VOC classes, including several SCFAEs, but it produced little anaerobic methanethiol compared to that produced by the other two species. This suggests that the anaerobic VOC profile of *C. subterminale* was more diverse than that of *C. sporogenes* and *R. lituseburensis*. In comparison, the aerobic metabolic profile was limited and similar among the 3 species, suggesting a constrained metabolic behavior for VOC production in the presence of oxygen. Unsupervised heatmap analysis (Fig. 4) confirmed the dichotomic metabolic switching since the atmosphere was the main discriminating factor and had a larger effect on the VOC profile than that of the species. Moreover, the accuracy of species identification was better when grown in anaerobiosis (perfect accuracy according to the unsupervised heatmap analysis, Fig. 4), and this was confirmed by discriminant analysis with 87.5% correct results for bacteria grown in anaerobiosis versus 75% for bacteria grown in aerobiosis after cross-validation.

### Butyrate production

**Figure 5:**
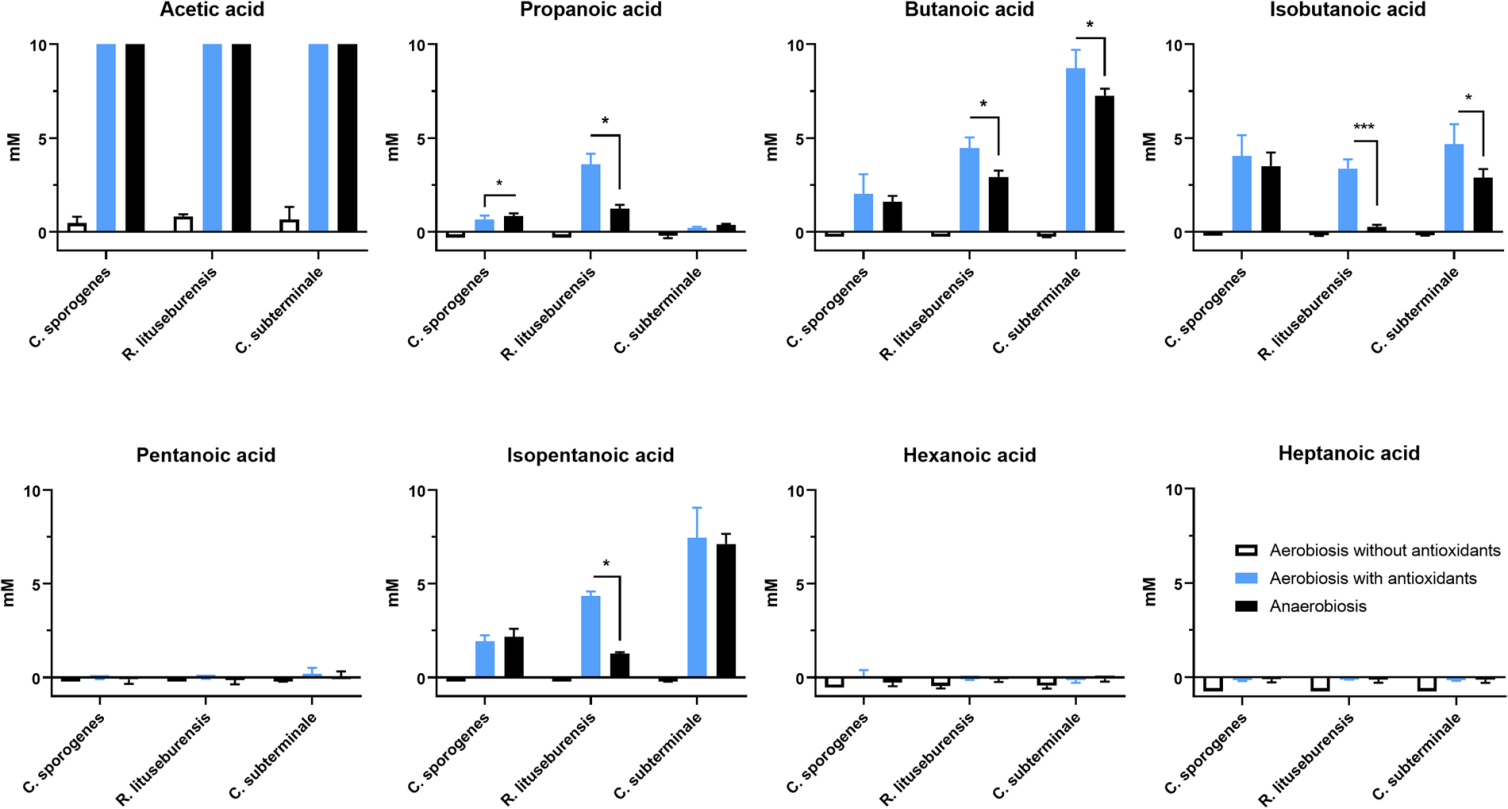
Comparison of short-chain fatty acids produced in antioxidant-based aerobic or anaerobic culture condition. Acetic acid (C2) was very abundantly (>10 mM: upper detection limit) produced by all strains in aerobic blood culture bottles supplemented with antioxidants, and in anaerobiosis. Production butanoic acid (butyrate), a key metabolite for human gut homeostasis, was higher in aerobiosis with antioxidants than in anaerobiosis. *p < 0.05, ***p < 0.0005.

We subsequently tested the production of nonesterified short-chain fatty acids in the presence of oxygen near the gut epithelium. Beyond the critical role of antioxidants in preserving gut epithelial-associated “anaerobic” microbes^17,24^, this could be a proof of concept for the critical role of gut antioxidant capacities to maintain the production of beneficial molecules by “anaerobes”, such as butyrate, under hyperoxygenation^25,26^. Very minimal acetic acid production was detected in vials of aerobic blood cultures without supplementation with antioxidants, while all other SCFAs appeared to be consumed even though the difference in concentration was very small (Fig. 5). Since none of the 3 bacterial strains survived growth in aerobiosis in the absence of the 3 antioxidant molecules, this could represent a transient metabolism before bacterial death. Pentanoic, hexanoic and heptanoic acids were not produced by any strain in any atmosphere. Acetic acid (C2) was very abundant (>10 mM: upper detection limit) produced by all strains in aerobic blood culture vials supplemented with the 3 antioxidant molecules and incubated in anaerobiosis. A significant difference according to both the atmosphere and species was confirmed by two-way ANOVA (p < 0.0001) for the contents of propanoic, butanoic, isobutanoic and isopentanoic acids with a very significant interaction. By comparing only bacteria grown in aerobiosis with antioxidants to those grown in anaerobiosis by multiple t-tests (one for each species) corrected for multiple comparisons, we were surprised to find that SCFA production was often higher for bacteria grown in aerobiosis with antioxidants than for bacteria grown in anaerobiosis (Supplementary Table S2). This was particularly significant for *R. lituseburensis* and *C. sporogenes*, which produced more isobutanoic than butanoic acid. *C. subterminale* did not produce a significant amount of propanoic acid but was the best producer of butanoic and isopentanoic acid. Indeed, it produced up to 8.7 ± 1.0 mM butanoic acid and 7.5 ± 1.6 mM isopentanoic acid when grown in aerobiosis with glutathione, ascorbic acid, and uric acid (Fig. 5 & Supplementary Table S2).

### Polar metabolites

**Figure 6:**
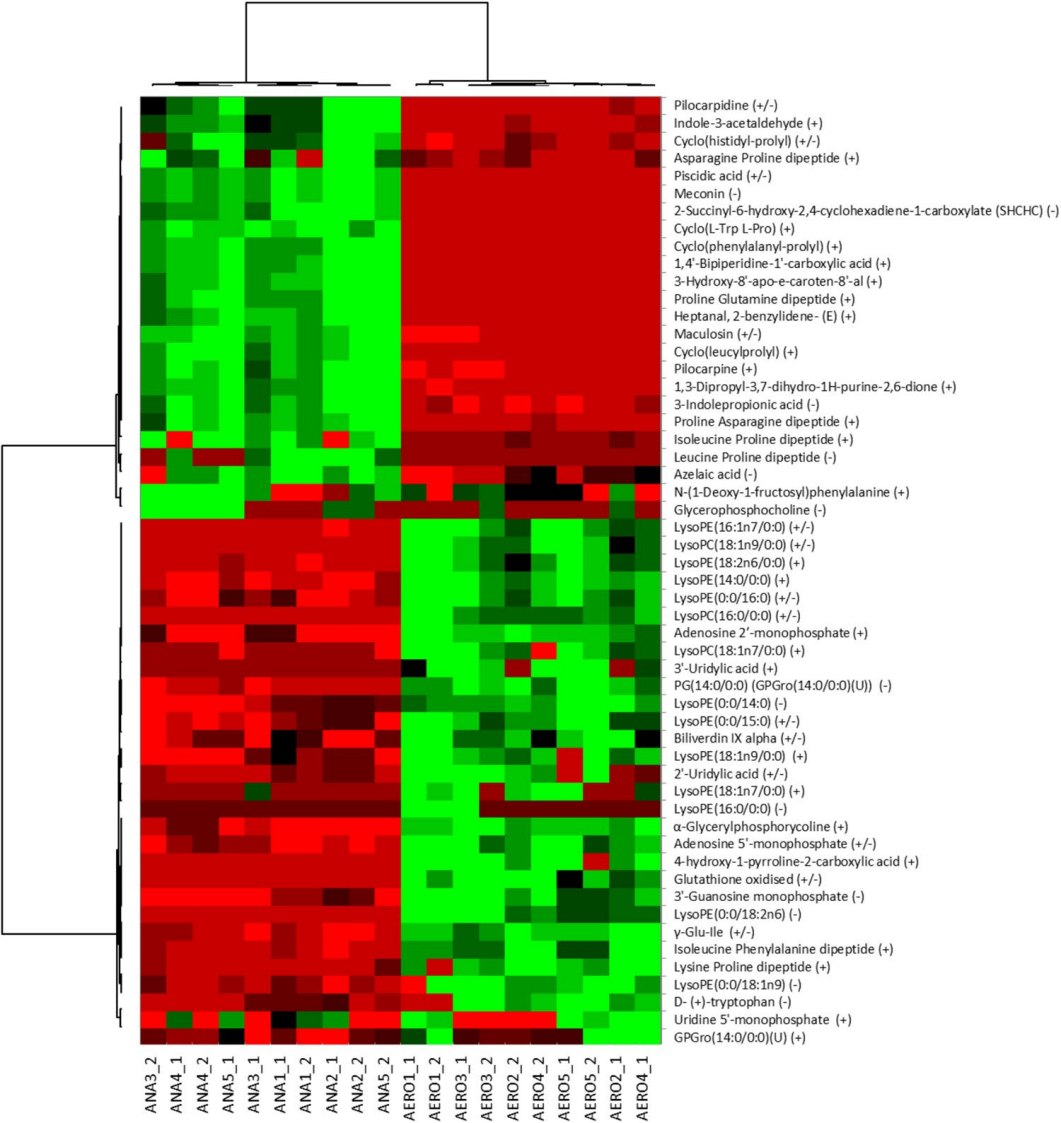
Polar metabolite profile according to atmosphere. The profile of polar metabolites was different in aerobiosis, with a major increase in the production of many lysophospholipids and some dipeptides. The detection of oxidized glutathione in aerobic conditions confirmed its antioxidant activity in the culture medium. ANA: Anaerobiosis, AE: Aerobiosis. This analysis was performed only for *C. sporogenes*. Measurements were performed twice for each of 5 experiments. Heatmap unsupervised analysis performed with XLSTAT v19.1.

By analyzing the production of polar metabolites (Fig. 6), we observed that there was a different profile for bacteria grown in aerobiosis than for those grown in anaerobiosis, with a major increase in the production of many lysophospholipids and some dipeptides. The detection of oxidized glutathione under aerobic conditions confirmed its antioxidant activity in the culture medium. On the other hand, pilocarpine, a parasympathomimetic specific for M3 muscarinic receptors that promote digestive peristalsis, was increased anaerobically. Some metabolites were specific to each atmosphere: 3′-uridylic acid, 4-hydroxy-1-pyrroline-2-carboxyclic acid and lysophophatidylethanolamine (18:1n7/0:0) were detected only for bacteria grown under aerobic conditions (Table S3). 1,4′-Bipiperidine-1′-carboxyclic acid, 3-hydroxy-8′-apo-E-caroten-8′-al, cyclo(L-Trp L-Pro), heptanal, 2-benzylidene-(E), leucine proline dipeptide, meconin, and SHCHC (2-succinyl-6-hydroxy-2,4-cyclohexadiene-1-carboxylate, precursor of vitamin K) were detected only for anaerobically cultured bacteria (Supplementary Table S4).

## Discussion

Here, we have highlighted a mechanism directly linking 3 main human antioxidants, anaerobic survival in the presence of oxygen and butyrate production. It is known that most butyrate producers in the human digestive tract are *Lachnospiraceae*, *Ruminococcaceae* and *Bacteroidetes*, which are anaerobic^27–29^. Antioxidants are known to keep anaerobes alive when exposed to oxygen or oxidative stress^16–20,24,30^. Here, we have shown that butyrate production is maintained or even increased by anaerobes even when anaerobiosis is lost. This was a specific mechanism because many other metabolites, such as methanethiol, sulfur alcohols and sulfur short-chain fatty acid esters, were no longer produced in aerobiosis with antioxidants.

The 3 molecules (glutathione, ascorbic acid and uric acid) were critical for *Clostridia* survival since no growth was observed in aerobiosis without these molecules^17^. The aerobic survival of strict anaerobes depends on the presence of molecules lowering the oxidation-reduction potential (redox potential) in the medium^20^, such as those containing an SH group^18^. This has been shown with alkali sulfides as early as 1898, pyruvic acid (1923), cysteine (1926), glutathione (1926), thioglycolic acid (1926) and many other thioacids (1928)^18^. As early as 1940, Brewer showed that the absence of trace oxygen was not sufficient for growing strict anaerobes and developed a medium based on sodium thioglycollate, D-glucose (dextrose) and agar^18^. Sodium thioglycollate lowers the redox potential, D-glucose is a carbon source for bacterial growth, and agar was critical for lessening convection (2). More recently, titanium(III) citrate (E°H = −480 mV) was also successfully used as a reducing agent for anaerobic growth, including the methanogenic *Archaea Methanobrevibacter arboriphilus*^20^. However, redox-lowering activity is not the only factor. Jones *et al*^19^. showed that titanium(III) citrate alone allowed for the slow growth of *Prevotella brevis*, *Butyrivibrio fibrisolvens*, and *Eubacterium ruminantium*. In contrast, the addition of cysteine hydrochloride, an antioxidant and sulfur source, increased bacterial growth by a factor of 100^19^.

Thioglycollate and titanium(III) have not been described in humans, while glutathione, ascorbic acid and uric acid are present and major antioxidants in animal and human plasma, gut and colonic mucosa^3,31–34^. The relative contribution to the antioxidant power of human plasma is 60% for uric acid, 15% for ascorbic acid, 10% for protein and 5% for alpha-tocopherol and bilirubin^35^. Glutathione is one of the major intracellular and mitochondrial antioxidants^36^. Uric acid is excreted in the gut and rapidly degraded by gut bacteria^37^. Further studies are needed to clarify the relative antioxidant power and role in microbiota maintenance of glutathione, ascorbic acid, uric acid, proteins, including glutathione-associated enzymes (glutathione-S-transferase (GST) and glutathione peroxidase (GPX)), alpha-tocopherol^38^, beta-carotene^38^ and gut bilirubin derivatives (urobilin, stercobilin and protoporphyrin)^39^ in the gut.

Beyond aerobic survival, we have shown the notable existence of an aerobic metabolic profile that is different from that of bacteria grown under anaerobic conditions. The mechanisms allowing for this change between the 2 metabolic profiles will have to be elucidated in future studies. Linares *et al*.^40^ reported that there is an inducible *ula* regulator capable of anaerobically catabolizing L-ascorbate in several bacterial species. An additional *YiaK-S* operon was required in aerobiosis. This use was associated with the production of short-chain fatty acids, such as acetate^40^. However, the fact that ascorbate or uric acid may serve as a carbon source for bacteria in the gastrointestinal tract does not preclude a potential antioxidant role. Moreover, the detection of oxidized glutathione confirms the antioxidant role of reduced glutathione in the medium. While butyryl-CoA:acetate CoA-transferase activity is dominant in butyrate-producing bacteria in the human digestive tract^27,29^, it is the butyrate kinase pathway that is present in *R. lituseburensis*^29^. Further studies should investigate the specific molecular mechanisms linking the presence of oxygen, glutathione, ascorbic acid, and uric acid in the stimulation of butyrate production.

The conservation of butyrate production in aerobiosis with antioxidants observed here is particularly important because butyrate itself limits the oxygenation of the digestive lumen; in colonocytes, oxygen consumption with oxidation of butyrate by colonocytes gives CO_2_ and ATP, and this is associated with water and sodium absorption^25^. Our findings shed light on the beneficial role of antioxidants on gut microbiota and butyrate production, previously reported *in vivo*, particularly with oligomeric proanthocyanidins (OPCs)^41,42^, but the microbial mechanism was not understood. Here, we show that antioxidants unravel an unexpected and unexplored aerobic metabolic profile of human gut *Clostridia*, and this profile is possibly active *in vivo* near the human gut mucosa. The physiological effect of the possible microbial production of alkanes, alkenes, and lysophospholipids near the human gut mucosa will need to be clarified in future studies. Nevertheless, the effect of butyrate on human gut mucosa and immunity is well known and is key to human health^3,5,6,43^.

Gut microbes are key and instrumental elements in the complex mechanisms linking environment, behavior, diet and human health^44^. Our study was an observational study and was designed to observe microbial metabolism according to the environment (atmosphere) and the presence of 3 major antioxidant molecules in the human body but not to clarify the molecular mechanisms. After linking gut redox and the microbiome^10^, this is the first study linking major antioxidants in the human body, gut microbiome and health through butyrate production. Future studies should investigate total microbial mass and metabolic pathways using biochemical analysis, proteomics, transcriptomics and genomics^40,45^ and include a control group grown in anaerobiosis with these 3 antioxidant molecules. It may be considered that the difference in the concentration of volatile organic compounds may also be related only to the difference in growth. However, this would only quantitatively and not qualitatively explain the differences in the metabolic profile.

Here, we demonstrated that a combination of 3 major antioxidants in the human body (glutathione, ascorbic acid and uric acid) allowed for human gut *Clostridia* to thrive and maintain microbial production of the key molecule butyrate in the presence of oxygen (in aerobiosis). This work provides new arguments for understanding the critical role of antioxidants in the maintenance of gut homeostasis and host-archaeal-bacteria mutualism and their resilience to uncontrolled oxidative stress and radical attack. This key role of antioxidants on microbiota probably extends beyond the digestive tract, as the beneficial effect of butyrate has been suggested for other human microbial ecosystems, such as the skin^46^, the gut-brain axis^47^ and atopic diseases^48^.

## Methods

### Culture conditions

Three strains of anaerobic bacteria, isolated by culturomics studies^21–23^ and obtained from the Collection de Souches de l′Unité des Rickettsies (CSUR, Marseille, France), were used for this study: *Clostridium sporogenes* CSURP3957, *Clostridium subterminale* CSURP3759 and *Romboutsia lituseburensis* CSURP3909. These bacterial strains were previously cultured anaerobically for 24 hours at 37 °C on Columbia agar supplemented with 5% sheep blood (BioMérieux, Marcy l’Etoile, France). Then, the bacterial inoculum was calibrated to 10^6^ bacteria/mL in PBS using a densitometer (Thermo Fisher, Villebon sur Yvette, France), and 0.1 mL of this solution (10^5^) was inoculated into different culture bottles using a syringe. Classical anaerobic culture conditions in standard anaerobic blood culture (Becton Dickinson, Le Pont de Claix, France) under a 100% nitrogen atmosphere were compared with aerobic culture conditions supplemented with an antioxidant cocktail in aerobic blood culture bottles (Becton Dickinson). Negative culture controls were performed under classical aerobic conditions in aerobic blood culture bottles (Becton Dickinson) without antioxidants. Sheep blood (5%) was previously added to each culture condition. The antioxidant solution was prepared as follows: a stock solution containing 4% ascorbic acid (VWR, Leuven, Belgium), 0.4% glutathione and 2% uric acid (Sigma-Aldrich, Lyon, France) was prepared in distilled water and filtered using a 0.2 μm filter after adjusting the pH to 7.5 with 10 M KOH. Then, 1 mL of this solution was injected into the aerobic blood culture bottle (Becton Dickinson) containing 40 mL of culture medium supplied by the manufacturer (for final antioxidant concentrations of 1 g/L ascorbic acid, 0.1 g/L glutathione and 0.4 g/L uric acid) using a sterile syringe and needle to avoid contamination. All blood culture bottles were incubated at 37 °C for different times (48 to 96 hours, see previous description) before measurement of VOCs, SCFAs and polar metabolites.

To confirm the viability of the bacterial strains at the end of the experiment, the blood culture content was subcultured anaerobically for 48 hours on Columbia Agar medium supplemented with 5% sheep blood (BioMérieux). Then, identification of the three bacterial strains was performed with MALDI-TOF-MS as previously described^49^. No viable bacteria were isolated after aerobic culture, while the viability and growth of all strains were confirmed in aerobic blood culture bottles supplemented with antioxidants and in anaerobic blood culture bottles.

### Volatile organic compounds (VOC) analysis

To measure the temporal production of VOCs in the bottles, different incubation times were tested for each culture condition: 48, 72 and 96 hours at 37 °C. After selecting the most suitable incubation time of 72 hours, VOCs were measured in triplicate for each condition. VOCs were collected for 10 minutes using a PDMS/DVB 65 μm SPME fiber (Supelco, Sigma-Aldrich, Saint-Quentin Fallavier, France) inserted in the headspace of each culture bottle. Gas and liquid chromatography-mass spectrometry (GC/MS and LC/MS) analyses were carried out with a Clarus 500 gas chromatograph equipped with a SQ8S MS detector (Perkin Elmer, Courtaboeuf, France) and with a liquid chromatograph. All data were collected and processed using Turbomass 6.1 (Perkin Elmer). A spectral database search was performed using MS Search 2.0 operated with the Standard Reference Database 1 A (NIST, Gaithersburg, USA). Chemical identifications were validated with both reverse and forward scores above 800. The measurement of a blank sample was carried out in parallel, consisting of a noninoculated blood culture bottle containing the same culture medium as the inoculated bottles. Consequently, the results only considered VOCs measured in culture samples after subtracting the corresponding blank values.

### Non-esterified short-chain fatty acid (SCFA) quantification

After finding that nonesterified short-chain fatty acids (SCFAs) were not detected by our first approach, we measured acetic, propanoic, isobutanoic (isobutyric), butanoic (butyric), isopentanoic (isovaleric), pentanoic (valeric), hexanoic (caproic) and heptanoic acids with a Clarus 500 chromatography system connected to a SQ8s mass spectrometer (Perkin Elmer) using the method described by Togo *et al*^50^.

### Polar metabolite investigation

*Clostridium sporogenes* was cultivated under the same conditions as described above. Five culture replicates for the anaerobic and aerobic antioxidant cocktail culture conditions were prepared, as well as their five respective culture controls (without bacteria). Collected cell pellets from 1 mL cultures were resuspended in 400 µL of methanol at -80 °C to quench the sample. After centrifugation, the supernatants were dried and reconstituted in 50 µL of 10% acetonitrile, 90% water and 0.1% formic acid. Pooled quality control (QC) samples were prepared by pooling all bacterial samples with equivalent quantities (culture controls were not pooled). Metabolites were first separated by reversed-phase chromatography using an ACQUITY UPLC I-Class System chromatograph (Waters, Saint-Quentin-en-Yvelines, France) and then monitored using a hybrid ion mobility QTOF mass spectrometer (Waters). Compounds were ionized in a Zspray ESI source using a default “Labile Tune” to reduce compound fragmentation before QTOF monitoring. Ionization parameters were set as follows: 0.8/2.0 kV for positive/negative modes, source/desolvation temperatures = 120/600 °C, and cone/desolvation gas = 100/600 L/h. Ions were monitored using a Sensitivity HDMSe data independent analysis method between 50 and 1000 m/z as follows: scan time, 0.15 s; collision energy ramp, 20–40 eV; and automatic mass correction during survey (using Lockmass 554.2620 m/z from a leucine enkephalin solution). Mass and CCS calibrations were run before analysis batches using the Major Mix solution (Waters). Raw data were then processed by UNIFI software to collect “components” described by retention time, drift time and precursor/fragment masses. Automatic parameters were set to detect 4D peaks above 150 and 75 counts for low and high energy scans. The marker table was sent to the EZinfo multivariate statistics software (Umetrics, Umea, Sweden) for data exploration by PCA and then data classification according to both anaerobic and aerobic groups using an OPLS-DA model. The selected markers were then labeled on the marker table in UNIFI and searched using the elucidate tool against the following structure libraries: metabolite structures (including CCS values, Waters) and the KEGG + HMDB databases (Chemspider access). Potential identities were then added to an inhouse structure library including.mol files from Chemspider (Royal Society of Chemistry, London, United Kingdom) and then targeted on the initial raw data according to the following parameters: a 5 ppm mass tolerance, a 2 mDa mass tolerance for predicted structure fragments, a 5% CCS deviation, and multiple adducts (+H, + Na, +K, + Li, +NH4) within 5 ppm and 0.1 minutes. Identifications were then filtered with the following parameters to validate metabolites: mass error <2 ppm for precursor ions, <2% CCS deviation (if available), at least one predicted structure fragment, <10% error on CCS estimates, <5 ppm error on RMS isotope match and <15% RMS percent isotope intensity^51^. The consistency of metabolites between positive and negative experiments were also checked (retention time, CCS values, and fold changes). The fold change was defined as the ratio of response values from the mean response for each condition.

### Statistical analysis

The peak areas of each detected VOC (arbitrary units) and the concentration of each SCFA (mM) were assessed as quantitative variables. Species (*C. sporogenes, R. lituseburensis* and *C. subterminale*) and atmosphere (aerobiosis without antioxidants, aerobiosis with antioxidants and anaerobiosis) were considered qualitative variables. Unless noted otherwise, three replicate values were measured. Comparing aerobiosis with antioxidants and anaerobiosis, three t-tests (one by species) were performed, and differences were determined using the two-stage linear step-up procedure of Benjamini, Krieger and Yekutieli, with Q = 1% (VOCs) or 5% (SCFAs). Each row was individually analyzed without assuming a consistent SD. When 3 groups were compared (aerobiosis without antioxidants, aerobiosis with antioxidants and anaerobiosis), two-way ANOVA was also performed with correction for multiple comparisons using Tukey’s test. Principal component analysis with Pearson metrics was performed, and biplots were shown to explore the association between strains, atmosphere and metabolites. Statistical tests were two-sided. A p-value ≤ 0.05 was considered significant. These statistical analyses were performed using GraphPad v8.0 (GraphPad Software, La Jolla, CA, USA) and XLSTAT v19.1 software (Addinsoft, Paris, France).

## Data availability

The data that support the findings of this study are available from the corresponding author.

## Supplementary information

Supplementary information.
